# Cardiovascular Complications of Anaplasmosis: A Case of Acute Pulmonary Embolism and Literature Review

**DOI:** 10.3390/idr18030062

**Published:** 2026-06-20

**Authors:** Aleksandar Gavrancic, Christian M. Jacobson, Veljko Rabasovic, Erik Sviggum, Jelena Stojsavljevic, Nestor G. Tarragona, Peter J. Mattingly, Igor Dumic

**Affiliations:** 1 Mayo Clinic Volunteer Program, Department of Hospital Medicine, Mayo Clinic, Jacksonville, FL 32224, USA; aleksandargavrancic@icloud.com; 2 Hospital Medicine Fellowship Program, Department of Hospital Medicine, Mayo Clinic Health System, Eau Claire, WI 54703, USA; jacobson.christian@mayo.edu (C.M.J.); stojsavljevic.jelena@mayo.edu (J.S.); 3 School of Medicine, University of Belgrade, 11000 Belgrade, Serbia; rabasovicveljko@gmail.com; 4 Department of Radiology, Mayo Clinic Health System, Eau Claire, WI 54703, USA; sviggum.erik@mayo.edu; 5 Division of Hospital Medicine, Lahey Clinic, Burlington, MA 01805, USA; nestor.g.tarragona@lahey.org (N.G.T.); peter.mattingly@lahey.org (P.J.M.)

**Keywords:** *Anaplasma phagocytophilum*, anaplasmosis, pulmonary embolism, thrombosis, tachycardia, emerging infections, ticks, tick borne disease

## Abstract

**Background:** Anaplasmosis is an emerging tick-borne infection that typically presents as a non-specific febrile illness, with variable degrees of cytopenias and liver tests abnormalities. Severe complications remain atypical and uncommon. **Case Report**: We report a case of acute pulmonary embolism (PE) occurring during confirmed anaplasmosis in a 73-year-old male with no traditional thromboembolic risk factors. The patient presented with fever, constitutional symptoms, thrombocytopenia, leukopenia, and abnormal liver tests, raising suspicion for a tick-borne illness. Despite early clinical improvement on doxycycline, persistent tachycardia triggered further evaluation and uncovered an acute PE. Comprehensive workup at admission and repeated 14 months later excluded inherited and acquired thrombophilias, malignancies, autoimmune diseases, and alternative infectious etiologies. The patient was treated with doxycycline 100 mg orally twice daily for 10 days and anticoagulation with unfractionated heparin followed by 6 months of apixaban for a first episode of provoked PE. He attained complete clinical recovery without recurrence of thrombosis at the two-year follow-up. **Discussion:** Infectious diseases are increasingly recognized as contributors to thrombosis through inflammation-mediated hypercoagulability and endothelial dysfunction. Pulmonary involvement in anaplasmosis typically manifests as pneumonitis, pneumonia or acute respiratory distress syndrome, but thrombotic complications such as PE are exceedingly rare. This case highlights a rare but clinically significant vascular complication of anaplasmosis and underscores the importance of considering thromboembolic events in patients with persistent or unexplained tachycardia. **Conclusions:** As the incidence of anaplasmosis continues to rise, greater awareness of its potential cardiovascular manifestations is essential. Early recognition and prompt treatment with doxycycline remain critical, while further studies are needed to better define the thrombotic risk associated with this infection.

## 1. Introduction

*Anaplasma phagocytophilum*, the causative organism of human anaplasmosis, is an obligate intracellular, Gram-negative bacterium that grows and replicates within *Ixodes* ticks and preferentially infects human granulocytes [1,2,3,4,5]. *A. phagocytophilum* shares its tick vector with several other well-described human pathogens, including the bacteria *Borrelia burgdorferi*, *Borrelia miyamotoi*, *Borrelia mayonii*, and *Ehrlichia muris eauclairensis*; the parasite *Babesia microti*; and *Powassan virus* [1,2,3,4,5,6,7]. Tick bites are the predominant mode of transmission, but vertical transmission, contact with infected blood, and blood or platelet transfusions are also recognized routes [2,3,4,5,6,7,8,9,10].

Human anaplasmosis typically presents with a non-specific febrile illness, and classical laboratory findings including variable levels of thrombocytopenia and hepatitis. Rare but serious complications such as acute respiratory distress syndrome [11] encephalitis [12,13], myocarditis [14], and HLH [15] have been described. Disease tends to be more severe in older and immunocompromised patients [1,2,3,4,5,6] and these patients’ characteristics are also associated with an increased risk for hospitalization [1,16].

Vascular manifestations of anaplasmosis are seldom reported. Only one case of pulmonary embolism has been reported to date, occurring in association with antiphospholipid antibodies [17]. There have been no reviews or retrospective studies on cardiovascular manifestations of the disease. We present a case of pulmonary embolism occurring in the context of anaplasmosis in a patient without other vascular risk factors, and contextualize this presentation through a review of the literature on cardiac and other vascular manifestations of anaplasmosis.

## 2. Case Report

A 73-year-old male farmer from Wisconsin presented to the emergency department with a one-week history of intermittent fevers, dizziness, nausea, and decreased appetite. He denied headache, chest pain, dyspnea, abdominal pain, or diarrhea. His past medical history was notable for chronic pain syndrome and migraine headaches, both well controlled with as-needed acetaminophen. He remained physically active and worked daily on his cattle farm. He had not recently traveled, had surgery, or experienced other immobility. He consumed alcohol socially, did not smoke, and denied illicit drug use. He owned a dog but reported no recent bites and did not recall any tick exposure, although he had substantial outdoor exposure due to his occupation. He denied any family history of venous thromboembolism.

Physical examination revealed an ill-appearing, weak elderly man with dry mucous membranes and decreased skin turgor. His mental status was normal. Vital signs demonstrated a heart rate of 115 beats per minute, respiratory rate of 20 breaths per minute, blood pressure of 95/60 mmHg, and normal oxygen saturation on ambient air. Electrocardiogram (EKG) showed sinus tachycardia without ischemia. The tachycardia was initially attributed to volume depletion. Laboratory evaluation revealed thrombocytopenia (platelet count 60 × 10^9^/L), normal hemoglobin (14.5 g/dL), and leukopenia (white blood cell count 3.5 × 10^9^/L) with lymphocytopenia. A comprehensive metabolic panel demonstrated hyponatremia (131 mmol/L) and mild transaminitis, with aspartate aminotransferase (AST) 79 U/L and alanine aminotransferase (ALT) 90 U/L, and a total bilirubin of 1.9 mg/dL with a normal indirect fraction. C-reactive protein (CRP) was elevated at 90 mg/L (reference < 5 mg/L).

**Figure 1:**
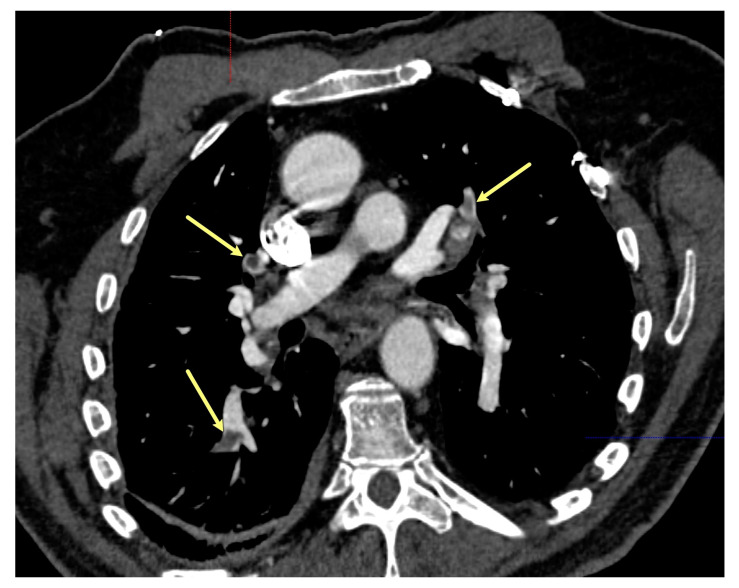
Obliqued axial view of the lungs demonstrates acute filling defects in the proximal right upper and lower lobe pulmonary arteries as well as the proximal left upper lobe pulmonary artery (yellow arrows) consistent with acute pulmonary emboli.

The patient was admitted to the hospital for supportive care including intravenous fluids. Given residence in a tick-endemic region, the pattern of fevers, thrombocytopenia, elevated liver tests, and constitutional symptoms raised suspicion of tick-borne infection, and empiric intravenous ceftriaxone (2 g daily) and doxycycline (100 mg twice daily) were initiated. Within 24 h, his clinical condition improved; however, he remained persistently tachycardic (115–120 beats per minute). Further evaluation revealed a normal thyroid-stimulating hormone (TSH) level and an elevated D-dimer of 2627 ng/mL (reference < 500 ng/mL). Computed tomography (CT) pulmonary angiography demonstrated an acute pulmonary embolism (Figure 1).

Thrombocytopenia had improved from 60 × 10^9^/L on admission to 71 × 10^9^/L on hospital day 2, and therapeutic anticoagulation with intravenous heparin was initiated. Prior to anticoagulation, a comprehensive hypercoagulability workup was obtained, including Factor V Leiden mutation, prothrombin gene (G20210A) mutation, antithrombin III activity, protein C activity and antigen, protein S activity and antigen, lupus anticoagulant (including dilute Russell viper venom time and confirmatory testing), anticardiolipin antibodies (immunoglobulin G and immunoglobulin M), anti–β2 glycoprotein I antibodies (IgG and IgM), homocysteine level, factor VIII level, fibrinogen level, Janus kinase 2 (JAK2) mutation testing, celiac disease panel, and paroxysmal nocturnal hemoglobinuria screening by flow cytometry and all results were negative.

Given the patient’s advanced age, malignancy-associated thrombosis was also considered. Prostate-specific antigen (PSA) was within normal limits, and CT imaging of the abdomen and pelvis revealed no evidence of solid organ malignancy. Serum protein electrophoresis and hematologic evaluation for lymphoma and leukemia were unremarkable. A screening colonoscopy performed one year prior had been normal and was not repeated.

Whole-blood polymerase chain reaction (PCR) testing was positive for *Anaplasma phagocytophilum*. Evaluation for co-infection, including *Babesia microti*, *Borrelia burgdorferi*, *Ehrlichia chaffeensis*, *Ehrlichia muris eauclairensis*, and *Borrelia mayonii*, was negative. The test was performed on EDTA-anticoagulated whole blood and molecular detection was performed using DNA extraction with the automated MagNA Pure 96™ system (Roche Diagnostics, Rotkreuz, Switcherland), followed by amplification of a conserved region of the *groEL* heat shock protein operon gene. Organism identification was achieved via melting curve analysis using the Roche LightCycler^®^ 480 II instrument (Roche Diagnostics, Rotkreuz, Switcherland) [18].

Following 72 h of doxycycline therapy, the patient demonstrated complete resolution of thrombocytopenia and marked clinical improvement. He was discharged with a plan to complete a 10-day course of doxycycline for treatment of anaplasmosis and a 6-month course of anticoagulation with apixaban for provoked pulmonary embolism in the setting of acute infection. At two-year follow-up, he remained in excellent health, with no evidence of recurrent thromboembolic disease, repeat hypercoagulable evaluation was negative, and there were no chronic complications from anaplasmosis.

## 3. Discussion

Infections are less common, but increasingly recognized risk factors for pulmonary emboli. They may promote thrombosis primarily by inducing a hypercoagulable state. Severe infections, particularly sepsis, are well recognized risk factors for venous thromboembolism, with reported odds ratios of approximately 1.4, increasing to as high as 10 in the presence of bacteremia [19,20,21,22]. Fat embolism may occur in severe sepsis as a result of bone marrow necrosis driven by systemic inflammation, ischemia, and coagulopathy [22,23]. Septic emboli represent another important mechanism and may arise in the setting of bacteremia, tricuspid valve endocarditis, infected central venous catheters, skin and soft tissue infections in persons who use drugs, and in patients with intracardiac devices such as pacemakers [22,23,24].

Thrombosis is established across multiple pathogens, including COVID-19 [25], varicella zoster virus (VZV) [26], *Fusobacterium* spp. [27], and malaria [28]. Among tick-borne diseases transmitted by *I. scapularis* in the United States, infection with *B. microti* has been linked to thrombotic complications such as splenic infarction [29,30]. Chronic infections, including tuberculosis, HIV, and hepatitis C, are also associated with increased thrombotic risk [31,32,33].

As an obligate intracellular pathogen, *A. phagocytophilum* evades the host immune response and promotes intracellular survival by suppressing the production of reactive oxygen species (ROS) within infected neutrophils, thereby impairing the oxidative burst [34,35,36,37,38,39,40]. However, despite this localized suppression, anaplasmosis induces a systemic inflammatory response characterized by the release of cytokines, including interferon-γ, interleukin-10, interleukin-12, and tumor necrosis factor-α [36,37,38,39,40]. In this regard, anaplasmosis mimics sepsis and COVID 19 infections which are also associated with cytokine storms. This inflammatory cascade not only compromises neutrophil antimicrobial function (explaining secondary bacterial infections following anaplasmosis) but also promotes oxidative stress in other compartments, particularly within the vascular endothelium. This compartment-specific imbalance in ROS may lead to endothelial dysfunction, characterized by reduced nitric oxide bioavailability, increased expression of adhesion molecules, and activation of procoagulant pathways. These changes contribute to endothelial activation and a shift toward a prothrombotic state through mechanisms such as tissue factor expression and enhanced platelet adhesion [36,37,38,39,40,41,42,43].

Another link between anaplasmosis and thrombosis stems from the neutrophils release of their extracellular chromatin, nuclear protein, and serine proteases to form neutrophil extracellular traps (NETs) [38,40,41,42,43] which entrap pathogens, induce endothelial dysfunction and trigger proinflammatory response, leading to coagulation. NETs promote thrombosis by providing a structural scaffold for clot formation [36,37,38] facilitating platelet adhesion, activating coagulation pathways and stabilizing the developing thrombus. Although direct evidence of increased NET formation in anaplasmosis is limited, the central role of neutrophils in both the infection and thrombotic processes suggests that NET-mediated mechanisms may represent a link between anaplasmosis and thrombosis. In susceptible individuals, this inflammation and redox dysregulation may be sufficient to trigger clinically significant thrombotic events, even in the absence of traditional risk factors for venous thromboembolism.

A prothrombotic state observed in *A. phagocytophilum* infection may also be explained by direct invasion of endothelial cells by *A. phagocytophilium*, leading to proinflammatory interleukin and cytokine production, endothelial activation, and increased expression of adhesion molecules. This endothelial activation may promote abnormal adhesion of erythrocytes and leukocytes to the vascular endothelium, contributing to a procoagulant microenvironment. Finally, *A. phagocytophilium* may cause platelet dysfunction by direct bacteria–platelet interaction or by indirect immune-mediated platelet destruction [44,45,46].

Pulmonary involvement in anaplasmosis typically presents as pneumonitis, pneumonia, and in severe cases, acute respiratory distress syndrome [11,34,35]. Thrombotic complications such as pulmonary emboli [17] are exceptionally rare and poorly characterized. In contrast to the case reported by Varwani et al. [17], which occurred in the presence of positive antiphospholipid antibodies, our case involved a comprehensive evaluation that excluded malignancy, autoimmune disease, and other comorbid hypercoagulable states. This supports a plausible association between thrombosis and anaplasmosis infection itself. Furthermore, following completion of six months of anticoagulation, there was no recurrence of thrombosis, and repeated hypercoagulable work up remained negative at the two-year follow-up. Whether thrombosis was due to anaplasmosis-induced endothelial injury as mechanistically highlighted above, or as a part of a cytokine storm as seen during sepsis remains unclear.

Other reported vascular complications in anaplasmosis include ischemic stroke [47,48] attributed to mechanisms similar to those discussed above. In addition to PE, the spectrum of cardiovascular manifestations in anaplasmosis includes myocarditis [49], myopericarditis [50,51], and atrial fibrillation [52]. New-onset arrhythmias are uncommon and most frequently present as atrial fibrillation, with or without concomitant myopericarditis [52,53,54,55]. Relative bradycardia and right bundle branch block have also been described [54,55], although they do not appear to have significant clinical consequences. In cases of suspected cardiovascular involvement, it is important to exclude coinfections transmitted by *I. scapularis*, particularly Lyme disease and *Babesia microti* infection, both of which are known to cause myocarditis and arrhythmias [55,56,57,58,59,60].

## 4. Conclusions

This case expands the evolving clinical spectrum of anaplasmosis by highlighting acute pulmonary embolism as a rare but potentially life-threatening vascular complication of anaplasmosis. Although cardiovascular manifestations remain uncommon, they are likely underrecognized and may become increasingly apparent with rising disease incidence and improved diagnostic awareness. This report underscores the importance of maintaining a high index of suspicion for thromboembolic and cardiac complications in patients with anaplasmosis, particularly when clinical features are atypical or disproportionate to the initial presentation. In this context, unexplained persistent tachycardia, even in the absence of chest pain or hypoxia, should prompt consideration of pulmonary embolism. Prompt recognition and timely initiation of doxycycline are critical for favorable outcomes. Anticoagulation should be initiated once pulmonary embolism is diagnosed, even in the setting of thrombocytopenia, provided there is no absolute contraindication such as overt bleeding. Further investigation is needed to better define the prothrombotic potential and cardiotropic effects of anaplasmosis.

## Data Availability

All data are available from corresponding author upon request.
